# eHealth usage among Chinese college students: qualitative findings

**DOI:** 10.1186/s12889-022-13521-1

**Published:** 2022-06-01

**Authors:** Hua-xuan LIU, Bik-chu CHOW, Chun HU, Holger HASSEL, Wendy Yajun HUANG

**Affiliations:** 1 School of Physical Education and Sport Science, Fujian Normal University, Fuzhou, 350117 Fujian China; 2 Department of Sport, Physical Education and Health, Hong Kong Baptist University, Hong Kong, China; 3 Student Mental Health Education Center, Northwestern Polytechnical University, Xi’an, Shanxi, 710000 China; 4 Institute of Applied Health Sciences, Coburg University of Applied Science and Arts, Coburg, Germany

**Keywords:** EHealth, EHealth literacy, College students, Health promotion, Web 3.0

## Abstract

**Background:**

The information technology has developed rapidly with the evolution of internet environment transformed from requiring computer skills for information searching to self-managing health data and applying information. Therefore, a more diverse range of eHealth skills is required and these skills are referred as eHealth literacy. However, most eHealth literacy studies focused mainly on information searching skills. Little is known about eHealth usage behaviors of college students in this day and age.

**Objectives:**

This study aimed to investigate how Chinese college students engage with eHealth tools and to determine the elements of their eHealth literacy.

**Methods:**

A purposive sample of 18 Chinese college students was recruited for in-depth interviews. Interviewees included three males and three females of each city (Beijing, Wuhan and Putian) from sports, medical, and non-health-related majors. Conventional content analysis was applied for data analysis.

**Results:**

The eHealth usage of different-major-students were compared and profiled by three themes of Expectance, Usage pattern and Perception. In Expectances, non-health major students applied eHealth only for urgent health need, sport major students used it to monitor health while medical major students, as frequent users for searching health database. In Usage pattern, purposes of eHealth for personal, practical and theoretical were identified for non-health major, sport major and medical major groups, respectively. In Perceptions, sport students felt more curious about eHealth than the other groups who perceived either fear (non-health students) or skeptical (medical students). By compiling those themes, the whole picture of eHealth usage was emerged. Based on that, the current study identified the related skills using the trilogy of Web 1.0 to 3.0, and derived a conceptual framework for eHealth literacy in the present day.

**Conclusions:**

The current study obtained a comprehensive understanding of eHealth usage and a framework of eHealth literacy required for Chinese college students. And it gives a clearer look at web 3.0 related eHealth behaviors.

## Introduction

eHealth was defined as ‘the information and communication technology (ICT) for health’ [1]. Interest in eHealth studies has grown substantially because of the rapid development in computer and internet technology innovation. Consequently, greater usage of health information technology is observed. Usage of eHealth has been shown to be a cost-effective way to access health information [2] and support health behaviors [3] for most people. However, because of the innovations in ICTs, the way people interact with eHealth is changing constantly.

To be specific, according to the most widely accepted generation division of internet evolution, the current internet environment has gone through three stages of development, namely Web 1.0, 2.0, and 3.0. Web 1.0 refers to the read-only web, while Web 2.0 refers to the read–write mode, providing a “social web” with greater collaboration and interactivity between consumers, programmers, service providers and organizations. Web 3.0, which is the current internet environment, refers to an integrated web where the machine will be able to understand and catalogue data in a manner similar to a human [4–6]. In the Web 3.0 stage, the previous way human interacts with machine (one-way tool-like approach) has totally changed. Yet, insufficient knowledge of how people make use of eHealth nowadays exists.

Previous research on eHealth usage has mainly been focused on online health information searching (Web 1.0), which is the major behavior of internet access [7, 8]. Generally, customers’ searching behaviors, like frequency [9–14], duration [13] and their frequently used device and website [15–20], are more likely to be discussed. However, general internet use is not effective in improving online health information searching [21]. Several scholars have noted that social media can provide additional sources of health information and encourage internet users to perform some participatory eHealth behaviors [22]. But few studies have focused on the health usage in social media by individuals [22, 23], and even fewer on the interactivities on the social networking sites (SNSs) (Web 2.0). With the fast-changing IT environment in recent years, newly developed e-tools are more integrated (one single device for multiple tasks) and immersive (linking the real world with the virtual one) [24]. Smart phone, as a new device to get internet access, has brought a huge revolution to people’s daily life and also increased the availability of health information [18]. However, the academic research on customers’ mobile internet usage with the exception of health information searching is scarce (Web 3.0). Hence, there is limited knowledge on the usage patterns of eHealth, especially the usage in Web 2.0 and 3.0 era.

In recent years, Chinese people have undergone a drastic media communication revolution. Near the end of 2018, 854 million people in China were internet users, and more than two-thirds of them used mobile phones [8]. It is essential for researchers and policy makers to obtain more information on eHealth promotion in China. It could not only benefit the health status of Chinese people but also provide valuable information on how to facilitate health service and communication in the context of rapid media and technological development. Since college students are among the most frequent internet users [25], it is worthwhile to investigate how they promote and maintain their health via the e-approach. It was reported that, with overall app use, younger and more educated users, like college students, are more likely to gain knowledge via internet or mobile internet [25]. College is an important transition time in people’s lives [26]. They may be making health decisions on their own for the first time and these could impact both immediate and long-term outcomes [27] including lifetime behavior patterns [28]. While numerous studies have shown the high mobile ownership among college students, a paucity of literature focused on college student usage of different e-tools (including website, SNSs and mobile apps) and eHealth tools.

Additionally, previous research showed that adults’ performance on using eHealth tools diversified by education and culture [17]; however, when it came to college students, few studies focused on the comparison between students from health and non-health majors. Only two articles compared the eHealth-related abilities between the health and non-health major students [29, 30], suggesting that specialized education programs could benefit students’ eHealth-related abilities. Yet both these articles did not provide information about college students’ eHealth usage behaviors. Moreover, with the popularity of fitness trackers and wearables, sport major students may have different eHealth behavior compared to other major students; as well, how sport major students interact with eHealth tools has not yet been investigated.

Moreover, updated eHealth tools called for a broader understanding of the capabilities and skills required for individuals to use and get benefited from eHealth service [31]. In 2006, Norman and Skinner labelled these capabilities and skills as “eHealth literacy” [32], with competencies being specified as to “seek, find, understand, and appraise health information from electronic resource and apply that knowledge to solving a health problem or making a health-related decision” [33]. However, with the advances in technology, a more diverse range of eHealth skills are required to fit in internet environment today [23, 33, 34]. Yet, there is insufficient knowledge in the newly required competencies of eHealth literacy, for instance, the capacity of dealing with mobile service, the sense of information safety, and the ability of tracking and managing personal health data, etc.

Addressing to these critical knowledge gaps, two research questions were raised: 1) how do people use eHealth tools nowadays? 2) what kind of abilities would be required while using eHealth tools? Accordingly, the primary purpose of this study was to profile Chinese college students’ eHealth usage behaviors, which including their expectance, experience and perception on different e-tools. Medical, sport and non-health major students were deliberately involved into the current study, for professional training might influence students’ health knowledge and eHealth skills, and by doing so, diversified views would be obtained. A secondary purpose was to identify the perceived related capabilities that are needed on eHealth usage (eHealth literacy). This exploration of eHealth usage behaviors could help with the development of a new measurement scale on eHealth Literacy.

## Research methods

This study was approved by the Research Ethics Committee of Hong Kong Baptist University. A qualitative approach was embraced in 2019 to explore the perspective, experience and perception of Chinese college students on eHealth usage.

To be selected as interviewees, participants had to 1) have the experience of using eHealth websites and tools; 2) have sufficient Chinese language proficiency to complete the interview; 3) have informed their willingness and consent of participating in the interview. Since disparities exist in China’s regional economic developments and cultural contexts and improved contextual uniqueness has an influence on individuals’ literacy [35], three Chinese cities, Beijing, Wuhan and Putian, were chosen deliberately for the recruitment of potential interviewees. The criteria for their selection were based on the cultural geographic (north, middle and south), the economic status (high, medium and low) and the administrative status (the capital of the country, the capital of a province, a prefecture-level city). Putian was deliberately chosen because it was well-known of its electronic commerce [36] and bad reputation on overpriced private clinical service^1^ [37]. It would be interesting to see if negative cases of eHealth information using (related to poor health literacy) could influence people’s usage on eHealth services and their attitude towards eHealth information. Furthermore, the choice of those three cities was also made with the issues of “convenience and feasibility” [38, 39] under consideration. After the target cities had been decided, the authors contacted with the lecturers or students who came from specific majors (sport-related major; medical-related major; and non-health-related major) of the universities in these cities. Then, a snowball sampling [40] approach was applied. Suggested by Burmeister and Aitken [41], data saturation is not about the numbers per se, but about the depth of the data. Although there is no definite criterion for assessing sample size appropriateness in qualitative research, the number of participants should be in accordance with the creed of theoretical saturation which means no new or relevant data seen to be emerging [42]. To achieve the theoretical saturation, based on the “rule of thumb”, the suggested number of participants for the interview studies is around twelve to fifteen [43]. In addition, a method to further enhance data saturation by including the interviewing of people that one would not normally consider could applied [44]. In previous studies, few scholars focused on including students of different study majors. Considering these two rules, in total, eighteen Chinese college students were recruited for in-depth individual semi-structured interviews over the phone. The consent forms were delivered to the participants via internet (using an instant message app, WeChat) before the interviews. Interviewees included three males and three females in each of the three major groupings (See Table 1). Therefore, the interviewees were “typical/representative” [39, 43] with significant comparability.

**Table 1:** Codes and demographics of interviewees

Code	Date (YYYY/MM/DD)	Length	Gender	Major	Region	Age	Current Year of Study
Interviewee 1	2019/01/02	28′40’’	Female	Medical	Putian	21	Year 2
Interviewee 2	2019/01/02	35′02’’	Male	Medical	Putian	23	Year 2
Interviewee 3	2019/01/02	24′59’’	Male	Non-health-related	Putian	22	Year 2
Interviewee 4	2019/01/02	30′15’’	Female	Non-health-related	Putian	21	Year 2
Interviewee 5	2019/01/08	43′50’’	Male	Sport	Putian	21	Year 1
Interviewee 6	2019/01/09	36′25’’	Female	Sport	Putian	21	Year 1
Interviewee 7	2019/03/15	30′21’’	Male	Non-health-related	Beijing	23	Year 4
Interviewee 8	2019/03/15	31′26’’	Male	Medical	Beijing	22	Year 2
Interviewee 9	2019/03/19	35′54’’	Female	Non-health-related	Beijing	22	Year 3
Interviewee 10	2019/03/21	52′21’’	Female	Sport	Beijing	23	Year 4
Interviewee 11	2019/03/25	45′59’’	Male	Sport	Beijing	23	Year 4
Interviewee 12	2019/05/21	39′10’’	Female	Medical	Beijing	21	Year 2
Interviewee 13	2019/05/12	63′33’’	Male	Medical	Wuhan	22	Year 3
Interviewee 14	2019/05/12	29′21’’	Female	Medical	Wuhan	25	Year 5
Interviewee 15	2019/04/06	47′27’’	Female	Sport	Wuhan	22	Year 2
Interviewee 16	2019/04/05	41′48’’	Male	Sport	Wuhan	22	Year 2
Interviewee 17	2019/05/11	30′59’’	Female	Non-health-related	Wuhan	22	Year 2
Interviewee 18	2019/05/10	31′13’’	Male	Non-health-related	Wuhan	22	Year 2

In order to achieve data saturation, interview questions were also structured with detailed elaboration questions to facilitate asking informants the same questions that guarantee a constant target [43]. The phone interview guide was designed based on the suggestions by Bryman [42] and Flick [45], as well as Hsieh and Shannon [46], in which the interview procedure starts with the establishment of rapport, introduction to the interview purpose and statement for the matters of process, and then continues with the main content of interview. The questions were open-ended and specific to the participants’ comments rather than to a preexisting theory. This is because the existing theory and research literature on eHealth is limited, by avoid using preconceived categories, those open-ended questions could allow the current study to inductively develop categories from the data [46, 47]. The interview guide included elements about individuals’ 1) previous experiences of interacting with eHealth tools; 2) reasons to use specific eHealth information and eHealth tools; 3) preference on eHealth information and eHealth tools; 4) common approach to interact with eHealth tools; 5) skills and strategies to obtain and apply eHealth information and eHealth tools; 6) attitude and perceptions on eHealth information and eHealth tools; 7) influence of eHealth usage on daily life; 8) outcomes of eHealth usage. Since the development of information technology was summarized into three phases (Web 1.0, Web 2.0 and Web 3.0), the interviews were structured following those three phases, for each phase questions were conducted around the eight main elements mentioned above. The interviews were audio recorded and transcribed verbatim. The transcribed scripts were returned to informants for verification. Each phone interview was conducted in Chinese (Mandarin) language and were recorded digitally with interviewee’s consent. Interviews ranged from 24 min to more than one hour. The detailed interview guide was listed below (see Table 2).

**Table 2:** Interview guide

Question	Elaboration questions	Theory, study or construct
**Did you search the Internet for health-related questions?**	-How often?	Description of use
	-Do you remember the last time you searched for a health issue?	Perceived susceptibility
	-What kind of information are you usually searching for, medicine, nutrition, fitness or daily care?	
**Could you describe a specific medical information that you had ever searched for?**	-Why did you want to search it?	Motive
	-How did you search it? (Which website/searching engine did you use? What keyword did you use? How to filter the information? How to find the answer? Could you understand it? Is the explanation clear on the Internet?)	Experience
	-Whether there is any communication with a third party (online doctor, online customer service) in the process? How is it? (understandable or not? did your problem be solved?)	Perceived behavior control
	-In this specific case, do you think the health information on the Internet can help you? Did your problem be solved at the end?	Ease of use
		Outcome expectation
**Could you describe a specific daily-care information that you had ever searched for?**	Similar to those of medical information	Similar to those of medical information
**How do you evaluate the health information online?**	-How do you determine if the information on the Internet is useful to you?	Perceived usefulness
	-How do you verify which information on the Internet is trustworthy?	Self-efficacy
**How do you feel about online health information?**	-How does it differ from offline health information?	Perceived benefit
	-Do you trust online health information? Why?	Perceived barriers
	-Under what circumstances would you search the Internet for health information	Self-efficacy
**Did you have conversation of health issues online with someone you know (family/friends)?**	-Did you ask them for help or suggestion on health issue?	Subjective norms
	-Did they ask you for help or suggestion on health issue?	Social support
	-If someone you know has a health problem and you happen to know how to solve it, would you volunteer the information?	
	-Did you take the initiative to share health information with your friends and family?	
	(why, how, perception & view)	
**Did you have conversation of health issues online with other netizens (strangers)?**	-Did you post online to seek help or suggestions on health issue? (which channel would you prefer, WeChat moments, Weibo, professional forums or popular forums?)	Self-efficacy
	-Did you reply to any help-seeking post online?	Visibility
	-Did you post online to share health related experiences, knowledge or opinions?	Social support
**Did you take use of e-consulting services?**	-What is it? How is it going?	Perceived severity
	-How do you determine if it is trustworthy?	Perceived usefulness
	-Did it fulfill your need? Why or why not?	Credibility
	-Do you find it an invasion of privacy?	
**Did you follow any health-related official account, key opinion leader or cyber celebrity on line?**	-Who? From when? For how long?	Uses and gratification
	-Did you actively browse the information posted by them?	Intention
	-Did it impact your health?	
**Do you use health apps?**	-what is it about? (medical/daily-care/fitness/nutrition)	Self-observation
	-which one is most frequently used?	Self-regulation
	-which one would you like to talk about today?	
**Why do you use this health app?**	-How did you find it? (peer recommendation or self-search)	Motives
	-Why you choose it? (compare with similar other ones)	Cues to action
	-What is the original goal? Which function did you make use of? Which function you do not pay attention to? Why and why not?	Social influence
	- What is the using perception? Easy-handling? User-friendly? Reliable or not?	Goal setting
	- How often did you use it? For how long? Would you continuously use it? Why or why not? (if uninstalled, why?)	Uses and gratification;
		Perceived ease of use
		Perceived usefulness
**Would you use health app to interact with others (social impact)?**	-Are your offline friends using this app? Do you think their usage will affect yours? What do you think of this kind of social function? Did it motive your usage? Did you pay attention to your friends’ post on the app?	Social support
	-Did the APP hold online activities? If so, do you think it is attractive? Did you take part in it?	Social influence
	- Did you make new friends by using health apps?	Visibility
	- Did you post on the APP? Did you forward your post to other social platforms (such as WeChat)? Did you share comments, experiences or photos on the APP?	
	-Do you find it an invasion of privacy?	
**Do you use intelligent health devices? Why do you use it? Would you use it to interact with others?**	Similar to those of health apps	Similar to those of health apps
**Do you use the health function in instant messaging (specially WeRun)?**	-Did you pay attention to the step-ranking and other users’ “thumb up”?	Uses and gratification
	-What do you think of this kind of function? Does it impact your health behavior?	Subjective norms
	-Did you go out of your way to do health behavior for your place on the ranking list?	Perceived social support
	-Did you use this function for health or for social interactions?	
**In order to better make use of the online health information, what kind of ability and knowledge should be obtained?**	-For website searching	Health literacy
	-For online inter-person communication	Self-efficacy
	-For public forums posting and reply	Competence feedback
	-For apps and intelligent health devices usage	
**What do you think of the different eHealth tools? (Web, social media, apps and intelligent devices)**	-The most frequently used one? The most fruitful one?	Perceived usefulness
	-What kind of role they are playing in people’s daily health maintaining?	Uses and gratification
**How much you care about your health?**	-Would you actively access health information in daily life?	Perceived susceptibility
	-If a friend of yours has been diagnosed with a serious illness, how will it affect you?	Perceived Severity

Data analysis was conducted by the conventional content analysis procedure [42, 46]. The transcripts were read word by word and coded separately by two PhD students with their research focus in the health promotion field. Codes then were sorted into categories based on how different codes were related and linked. According to Hsieh and Shannon’s [46] suggestion of inductive category development, knowledge generated from the procedure of content analysis should be based on participants’ unique perspectives and grounded in the actual data. Thus the coders allowed the categories to flow from the data [47]. When the two coders finished their own category-identification, the two sets of categories were compared and contrasted, and consequently grouped into possible themes. Then a back-checking between the themes against the data was constantly conducted. The consensus on the final themes and constructs was reached through discussion among the two coders. According to Brod et al.’s suggestion [48], having a second party (coder) to conduct the coding of transcripts could ensure reaching data saturation.

The two coders involved in the current study both have Chinese ethnicity. The coding and analysis were conducted in Chinese to be in accordance with the cultural circumstances, while back translation was adopted to the themes, categories and some original quotations. The lead author, an English language major in undergraduate studies was responsible in translating Chinese scripts into English language.

A number of techniques were applied to maximize trustworthiness of the data [49]. The scripts were reviewed to see if there were any inconsistencies in an interviewee’s responses, and categories were supported by quoting participants’ original words. Moreover, during the data analysis, the two coders agreed with each other 92% of the time (256/278) before reconciliation and 100% after reconciliation, which, referred to a good level of intercoder reliability [50]. Additionally, a triangulation between the findings (results related to Web 1.0 and 2.0) and related previous theories was applied to verify the reliability of results and the attainment of data saturation by ensuring that data is rich in depth [51]. Following data analysis, it was found that in the current study, there was enough information to replicate the study (one of the coders randomly selected 9 informants and recoding the data, found the results of the recoding could be consistent with the current), the ability to obtain additional new information has been attained, and further coding is no longer feasible. In that case the authors of this research determined that the data saturation had been reached and no further recruiting for informants were needed [51].

## Results

**Fig. 1:**
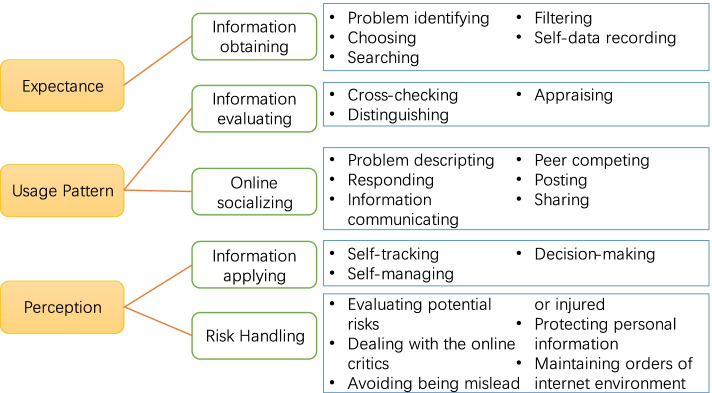
Themes and sub-themes of the current study

Qualitative data were organized through interviews and analyzed by conventional content analysis method. At the very first stage, data of students from different majors were mixed up to be coded, through which the whole picture of eHealth usage across college students from different majors was obtained. The whole picture of eHealth usage included five elements: information obtaining (i.e., problem identifying, filtering, choosing, searching, self-data recording, etc.), information evaluating (i.e., cross-checking, distinguishing, appraising, etc.), online socializing (i.e., problem descripting, responding, information communicating, peer competing, posting, sharing, etc.), information applying (i.e., self-tracking, self-managing, decision-making, etc.), and risk-handling (i.e., evaluating potential risks, dealing with the online critics, avoiding being misled or injured, protecting personal information, maintaining orders of internet environment, etc.). Afterwards, we looked into the obtained information (i.e., the eHealth usages) and found the diversity of different major students. The diversity was verified by the two coders as well as a researcher who helped review our data analysis as a ‘critical friend’ [52]. Therefore, the current authors decided to separately profile the eHealth usage of students from different major, and present the result in the current way. Three themes were identified to provide insight into college students’ eHealth: the Expectance, the Usage pattern, and the Perception on eHealth usage. The Expectance referred to information obtaining behaviors, for behaviors mentioned under this sub-theme were driven by specific expectances; the Usage pattern was linked to the evaluating and socializing behaviors; and the Perception referred to behaviors of applying and risk-handling, this is because different perceptions on eHealth tools oriented students to apply eHealth information in different degree, and to have different focus or worries about the potential risks (See Fig. 1).

The eHealth tools used widely at present were also identified through interview data (see Table 3). In general, they could be categorized into three main segments: 1) the traditional web-based read-only (or mainly for reading and receiving information) tools (web 1.0 tools), 2) the ones with social networking function to enable users’ interpersonal communication (web 2.0 tools), and 3) the ones based on mobile internet technology, collecting personal health data and provide personalized services (web 3.0).

**Table 3:** The most widely used eHealth tools

IT generation	eHealth tools
Web 1.0	Search engine
	Academic database
	Key opinion leaders & official accounts
Web 2.0	eHealth consulting platform
	Forums
	Instant messaging
Web 3.0	Sport function in instant messaging (specially *WeRun*)
	eHealth APP
	Intelligent eHealth devices

### Expectance—stopgap vs monitor vs database

It was found that, students from different majors have different attitudes toward their health. Those attitudes orienting them to have different expectances for the existing eHealth tools.

#### Non-health major students – stopgap

For six students from non-health major, five of them admitted that they cared less on health because they did not perceive it severe. They also thought that daily-care behaviors such as skincare or dieting were not counted as “health managing”. Most of the time, they held a primitive knowledge on the definition of health, which is equal to “not getting sick”. The non-health major students reported that they might engage health behaviors for body-shape-building, entertainment, or social purpose, but usually health was not the primary concern. Even about personal health caring, they were not sure what and how to do.

“I won’t pay attention to health issues until I am troubled by it.” (Interviewee 3).

Because of their attitude on health caring, half of the non-health major students seldomly interacted with eHealth tools until they had health issue. Interviewee 4 and 9 said that sometimes they would get fragmented daily-care information from influencers they followed, but both of them were passive recipients, not yet actively seeking opportunities to access health information. Meanwhile, the non-health major students did not fully believe in what they obtained online, in that case, they usually took eHealth tools as a stopgap – a tool that serves their urgent or temporary health purpose for a short time. The eHealth tools were expected to provide a “general picture” rather than the exact answer. The non-health major students were found to mostly rely on search engine to solve their health problems.

#### Sport major students – monitor

All six sport major interviewees reported that they have been conditioned strongly to their very physical active life and got used to pay attention to manage their health. They perceived that the requirement of their major and the environment of their school did impact their attitude and behaviors on health maintenance, especially physical activity participation. Specifically, two of them reported that they perceived being sub-healthy (a state between healthiness and disease) those days (interviewee 11 perceived overweight and 15 perceived sleeping-late), and they were actively adjusting their daily routine.

Corresponding to their attitude in keeping health, half of sport major students had browsed through the posts of fitfluencers at their leisure actively. Every one of them reported consciously making use of health apps. This is because the Web 3.0 tools, such as health apps and intelligent devices, provide the service for users to “real-timely” track and quantify their own health behavior and body data. Therefore, students usually used eHealth tools as the monitor of their own health behavior, by which students perceived more convenience in self-health-management and gained greater insight into their own fitness indexes. Although some index or information might be inaccurate, the sport major students still found those apps helpful.

“I want to know my heart rate and pace, although it may not be accurate, the data provides me the sense of security and control.” (Interviewee 16).

#### Medical major students – database

The medical major students showed high intention in keeping health similar to the sport major students, but in a medical way. They all had tried to self-treat some minor ailments. Moreover, they all reported that they had accessed to health information actively. Interviewee 1 and 14 said it was driven by requirement of the major, while interviewee 13 also mentioned about the responsibility of being a future medical doctor in practice. Four of them claimed that they often took the medical information online as references when solving health problem. Three informants said they had browsed the eHealth consulting platform for studying actual cases. The eHealth tools were treated as the databases by the medical major students. In addition to that, it was reported that the smart push technology in different apps, forums, and websites had pushed related health information to them based on their reading tendency.

“Being able to get access to online health information provided me a sese of security.” (Interviewee 13).

Compared with the sport major students, the medical major students interacted with Web 1.0 eHealth tools more, but seldomly used the Web 3.0 ones. Five of them declared that they rarely pay attention to other users’ records on health apps or WeRun, and their own exercise routine had never been impacted by the records. Meanwhile, two of them used health apps to record their exercise patterns and menstrual cycles, and both took their records as references to managing their health.

### Usage pattern—personal vs practical vs theoretical

#### Non-health major students – personal

As mentioned previously, non-health major students mainly use eHealth tools to solve a specific health problem, their usage pattern thus worked in a personal way, trying to find the information targeted to their health issues.

##### Information evaluating

Compared to the other two major groups, when searching health issue online, some of the non-health major students (interviewee 4, interviewee 17) perceived difficulty in identifying keywords, and rarely (2/6) knew any trustworthy web sites for searching in-depth health information. Since the reliability of online health information is uncertain, they showed preference on the information provided by famous people, which included some influencers. For the doubtful information, most non-health major students chose to skip them. The writing style of information would also affect their choice.

“If the headline (web title) is too dramatic or cliche, I would definitely not click in.” (Interviewee 4).

After narrowing the scope down, the credibility and feasibility of health information were subsequently judged. The judgement was made based on common sense (3/6), experience (3/6), and sometimes intuition (interviewee 4). Only interviewee 7 and 18 mentioned about checking different websites to verify the information. For the online-to-offline cross-checking behaviors, half of the six interviewees had consulted offline professionals for uncertainty online health information. None of the non-health major students had ever done online verification for the health information provided by offline professionals.

##### Online socializing

Most of the non-health major students (except interviewee 7 and 9) said they never used eHealth tools to socialize with strangers or make new friends. They usually used the tools to interact in a small scope, for instance, close friends or family, kept it in a personal way.

“When using the APP, I will only follow the friends I know in the real world.” (Interviewee 4).

As new users, students from the non-health major preferred to transfer their existing relationships to eHealth tools rather than build up a new network from scratch. Even for those who were willing to build a new eHealth-based network, the relationship was usually very fragile unless being transferred to some popular instant messaging sites (i.e., WeChat or QQ).

“I know someone on a forum who is very knowledgeable in body-building. After we became friends, most of the time we contact each other through WeChat. All in all, instant massaging is not the core function of the forum.” (Interviewee 7).

#### Sport major students – Practical

Unlike the non-health major students, the sport major ones performed a practical pattern in eHealth usage. Compared with the other two groups, the sport major students could be categorized as active tryers and social players.

##### Information evaluating

Most sport major students believed that knowledge could come from practice. Half of them reported that they had tried the online health suggestions several times. Interviewee 16 expressed that his trust on eHealth tools sometimes were built on the basis of previous effective trial usage.

“Some of my peers had searched online for better weight-control, then did not even have a try. It is meaningless. People cannot lose weight by just reading.” (Interviewee 10).

Compared to the non-health major students, the students from sport major showed stronger demand for information of higher quality. Two informants said they would use specialized eHealth apps as an ‘advanced’ searching engine to obtain information. The sport major students were also the only group who showed willingness to pay for better information (3/6). Meanwhile, they demonstrated the strongest intention to try eHealth tasks with reward provision (i.e., subsidy or opportunity of doing charity).

“I bought a critical illness insurance, the insurance company promise that if I can keep 10,000 steps per day, I could have a discount on the insurance fee. And I am working on it to save money and keep healthy.” (Interviewee 16).

Sport major participants were clear about their purpose on tool-using. For better satisfying their health needs, some of them (2/6) used several apps of the same type simultaneously to fit their needs (i.e., use JoyRun and NikeRun at the same time), while some would expect e-tools to play a supplementary role.

“It is fine enough as an assistant…But I won’t fully rely on it to help me do my specialist training.” (Interviewee 10).

##### Online socializing

Compared with the other two groups, sport major students’ usage of eHealth tools was not only more active, but also more sociable. All of them reported that they had used eHealth tools to socialize with others. Besides, they were much more willing than the other groups to post personal record online. Interviewee 15 admitted that the motivation for posting her personal records was intended for social interaction rather than self-recording because it made her visible to the public. Some of the sport major students also perceived that online interpersonal eHealth communication could provide novel ways for social interaction. Specifically, they mentioned that with the help of web 2.0 tools, relationships were created and strengthened, or getting healthier.

“One of my friends has a feeling on a girl but he is too shy to express it, and every time when this girl posting her jogging map tracking, my friend would give a thumb up to kind of give a signal.” (Interviewee 5).

Sometimes, students from sport major perceived more comfortable in communicating health behavior with strangers than with their family or peers.

“My APP usage records will be posted on Weibo because most of my followers on Weibo are strangers…I feel a little uncomfortable to post my physical activities to my friend in real world.” (Interviewee 10).

Beyond eHealth-based socializing, the sport major students’ usage of eHealth tools was more likely to be influenced by their social network. The interview data showed that eHealth-based relationship (i.e., offline relations who have similar health concerns, or new friendship made via eHealth tools) deeply influenced the sport major students’ choice on eHealth tools, and their health decision-making as well. Even the comments or recommendation from other users (who the interviewees may never have been in contact with) would have a strong impact.

“I know that the app ‘go ski’ is more professional and could track my movement better, but most of my ski-friends are using ‘Huabei’. In order to have more fun to go ski with them, I am using ‘Huabei’ instead of ‘go ski’.” (Interviewee 11).

“When purchasing new eHealth tools, I would visit some fans-forum and check other buyers’ comments and perceptions. The software data is difficult to understand, but users’ comments not.” (Interviewee 16).

Beside of the fruitful contents created by strangers, the number of strange users, which means the popularity, was also an important indicator for the sport major students to use eHealth tools. All six sport major students reported their willingness to reply another netizen/stranger’s post on health forums, and said they were not hesitated to seek help online if needed. Two of them had posted their health issues on apps and forums for seeking help. Four of them had tried the online health suggestions.

“I prefer to choose the platform with more users, so there would be more information in that platform.” (Interviewee 10).

#### Medical major students – theoretical

With expertise in health knowledge, most of the medical major students were skillful information accessor, frequently obtained in-depth health information online. However, they seldomly socialized online. A theoretical eHealth usage pattern was found existed in this group.

#### Information evaluating

##### Information evaluating

All of the six medical major interviewees knew several trustworthy web sites or apps for checking health information, and they all claimed that they had ever double-check uncertain health knowledge via the internet. They were also very picky on the e-source of the health information. Four of the six medical-major students expressed that they would search more in-depth health information through academic databases, medical forums, disclosed online doctor-consulting when encountering a health issue. Two participants (Interviewee 12, 13) mentioned that the interaction among users may also influence the quality and future development of the health information sources. Interviewee 18 specifically explained that he often checked the qualification of information providers, even he was browsing a qualified e-source.

“On Dingxiangyuan (a Chinese professional forum only accessible for medical practitioners) there would be detail descriptions and cases, and doctors would explain how they diagnose the patients. Those are very helpful, and hard to get through searching engine.” (Interviewee 9).

The medical major students showed skillful filtering strategies during interview, including to directly skip the information with advertising tag, to browse the page excerpts first to exclude the irrelevant ones, and to preferentially view the information provided by qualified sources (e.g., Baidu Dataset and Doctor consulting platforms). Those strategies helped them improve the efficiency of information searching, while not to miss the helpful ones. Interviewee 8 also perceived that assessment reports published online more trustworthy than other netizen’s comments and experience, even if it was provided by the eHealth service provider, which, contrasted with some sport major students’ opinion.

Besides, the medical major students were the only group that specifically mentioned lots of triangulation ways to verify the reliability of information – they not only cross-checked the information among different eHealth websites and tools (Online-to-online checking) (6/6), but also validated online information from offline professionals (Online-to-offline checking) (mainly discussed the online clinical cases with classmates) (4/6). Moreover, they all had sought online information to validate offline ones (Offline-to-online checking).

“I once had helped my family to check doctors’ advice online because I want to make sure the doctor had told us every side-effect.” (Interviewee 1).

##### Online socializing

The medical major students were inactive in eHealth-based communication, except with acquaintances. Four of them admitted that they often shared health information via internet within acquaintances that they trusted. They felt good when using their knowledge to help people they care without considering the limitation of time and space. Interviewee 13 told the interviewer that, when sharing health information, he preferred to forward the link directly to others rather than explain it in his own words, so that the information transferred could be more accurate and avoid the misunderstanding. Also, interviewee 2 said that he would avoid any recommendation for specific drugs or tools when sharing. All six respondents reported that their focus of health information could be influenced by acquaintances, for example, if acquaintances come to them for seeking health-related help, they would pay extra attention on the related field and rigorously help find the related information.

### Perception—fear vs curiousness vs skepticism

#### Non-health major students – fear

When talking about the perception on using eHealth tools, the students of non-health major expressed their fear on being misled, being criticized and personal data being leaked.

##### Information applying

All the non-health major students had expressed their concern about the credibility of eHealth information and their limited confident in evaluating eHealth information. Four of them mentioned that they perceived barriers in finding the “exact answer”. All of them showed a degree of distrust on online health resources, and admitted that negative news on information credibility would influence their attitude toward online health information.

“I always have uncertainty in evaluating the credibility of health information, thus I often with low sense of security when applying eHealth information.” (Interviewee 17).

Nevertheless, the non-health major students took online searching as the first choice whenever they are facing health issues. As mentioned before, they were not expecting the exact answer. In that case, most of them (4/6) perceived the existing eHealth tools were fit to their expectation and helpful enough, while the other two (Interviewee2, 7) agreed that the eHealth tools were playing a supplementary role in daily life for it usually cannot fully fit their health need, and sometimes troublesome to use.

“Mobile phone is a boundary for me when doing exercise. With it I cannot move comfortably, I may worry about drop and break it.” (Interviewee 7).

##### Risk handling

Considering the risk of misled by online health information, the non-health major participants indicated that they tend to trust and have a try on the daily-care information or fitness ones because of having low risk to try them, while dare not to trust the clinical-related ones – being perceived as the high risk makes it unworthy for the respondents to have a try.

“I think knowledge of health maintenance has no risk. Even if we mis-used it, or it is a fake one, this kind of try would do no harm to health. Thus, I feel it is fine to try or share it.” (Interviewee 18).

When it comes to the clinical issues, most students from the non-health major (5/6) perceived that the offline information was more trustworthy than the online ones. Two non-health major interviewees (Interviewee 1, 9) mentioned about the perception when facing the online critic and even trolls, which, the authors had not yet found in previous academic articles.

“I won’t actively share health information, because my friends and family may doubt my ability and show distrust, I am a little scared of being denied…I guess the ability of handling online critics should be acquired for every internet user”. (Interviewee 1).

Participants also mentioned the concern about data security. To make use of the eHealth service, internet users must provide some personal data, which may lead to personal data disclosure. All the non-health major students were aware of this risk, but only interviewee 17 held the idea that users should be extra careful when providing personal data, all the others showed a “let-it-go” attitude, felt that the leakage of personal data was inevitable.

“If you choose to accept the service, you must provide your personal data, otherwise the app cannot be used. For example, if I order a meal online, I have to enter my phone number and my address, and then, my important personal data leaked. I think I may just accept it and stop worry about it.” (Interviewee 7).

#### Sport major students – curiousness

Sport major students had a positive perception on their knowledge on health and their ability to control risks, so most of the time they are curious about eHealth tools and information.

##### Information applying

It was found that the perception of novelty motivated the sport major students to try new eHealth tools, although the interest may not last long. Four of them admitted that they would love to try novel devices related to health. Two participants bought new intelligent devices because of novelty. Additionally, all of the sport major students reported being literate in their self-health-data collected by the health apps, and perceived being able to make use of the personal data or exercise record.

For the credibility of eHealth information, it was reported not being a problem for sport major students. Five of them said that they had the proper e-channels or persons to consult for health issues (e.g., clinic, pharmacy, a medical major student, coach in a gym or their teachers). They also had confidence to evaluate the health information and apply them well. Half of them said that negative comments on eHealth information credibility could not influence their confidence. About the accuracy of eHealth tools/devices, they perceived it was acceptable for general public.

In addition, the sport major was the most warm-hearted group in responding to help-seeking requests online. They perceived that a fruitful interaction between online “hobby friends” could raise a higher demand toward the professionalism of health information, and may promote the development of knowledgeable forums for health and sport.

“When I was firstly fond of doing gym in 2012, there was little channels for me to know more about it, but now there are thousands of fitness apps and fans forums to self-learn and discuss it.” (Interviewee 11).

It is worth to mention that Interviewee 16 also admired the anonymity of eHealth tools.

Meanwhile, sport major students expected further improvement of eHealth tools. Interviewee 11 sometimes posted his training plan online, but was bothered by plagiarism. Thus, he specially insisted on the originality of ideas and information, expected a no-plagiarism-or-misappropriation environment could be built in the near future. Meanwhile, Interviewee 10 expected the eHealth tools could be developed for more sports, for example, basketball or table tennis, and a relevant index, such as reflex and movement speed, could be collected.

##### Risk handling

It should be mentioned that the students from sport major reported trying on the health information related to sports only. Although informants from this group were suggesting the bravery of taking action, the ability of controlling risk when trying was specially mentioned. They explained that their dare to undertake the risk of trying something new was because they had the confident in controlling their muscles to avoid injury and recognizing the fake health information. The self-cognition on health and ability was suggested to be continued through the beginning of information access to the very end (Interviewee 6), so that users could adjust the application on time according to the changes of physical condition.

“I felt that some beginners would follow the online suggestions blindly and easily trust some cyber celebrities, that may lead to some potential health risk.” (Interviewee 15).

The sport major students were not troubled by the personal data security issue. Some of them felt that those data exposed by the eHealth tools were nothing serious even if they were leaked. Some thought posting personal information should be an individual’s choice and one had the right to do so.

“Sometimes you just want to post some of your personal data, for example, your running record. Although that may cause threat, I guess we should respect this kind of behavior”. (Interviewee 6).

#### Medical major students – skepticism

The students from the medical major expressed the motive of self-protection regarding to the eHealth environment from potential criticism or abuse, and had a cautious view on eHealth usage. The current research outlined it as skepticism.

##### Information applying

None of the medical major students perceived that health information on websites had met their needs. They felt that the online health information was mostly inadequate in freshness. Interviewee 2 pointed out that it was usually hard to find the newly updated cases or the earliest ones online when it came to a not-generally-recognized disease. Also, eHealth tools were accused of being lack of specificity or professionalism. In addition, the credibility of health information on the web or collected by devices was questioned. Interviewee 12 specially mentioned that health information from qualified sources might also be misunderstood and would lead to some adverse impact on health. In that case, the medical major students suggested every eHealth user should obtain some common knowledge on health, find the trustworthy resources which was comparable to the users’ knowledge level, and build up the ability of cross-checking. Half of them believed that the truth or the right answer can be organized through rigorous verification, while the other ones suggested people with lower health literacy need to keep contact with one or two experts in health.

Although the medical major students were suspicious on the quality of eHealth information environment, their self-efficacy in evaluating online health information was persistent. All of them expressed that the negative views on information credibility would hardly affect their attitude toward online health information.

“I feel like that people with certain knowledge of health will have their own judgment and be less affected by varies information, for example, the news of Putian Hospital^1^ did little impact on me.” (Interviewee 12).

##### Risk handling

As mentioned before, the medical major students were skeptical about being active online. They showed strong awareness on self-protection and mostly had a state of alert when giving advice or posting personal data. Most of them (4/6) indicated that they rarely shared or posted their own data online. Two of them (Interviewee 12, 13) thought it might cause potential safety risk, for example, provide location information for possible stalkers. All of them were very mindful when sharing health information or suggestions online and admitted that they would not volunteer giving health information to other strangers.

“I rarely post or share information online for the purpose of protecting myself…most of the medical-related issues is serious, I don’t want to make mistakes or get into trouble.” (Interviewee 2).

Two respondents (Interviewee 8, 13) explained deeper about the reasons why medical students expressed cautious on sharing health information. The first one was that most people could not describe their symptom correctly and in detail, which probably might cause misdiagnosis.

“It is very hard to give suggestion if the help seeker online doesn’t know how to describe his/her symptom, for example, how is the pain feels like? Is it sharp or dull? Is it persistent or intermittent? But most people cannot tell it like this.” (Interviewee 13).

The second reason is that, in recent years, doctors in China have been abused, injured, and even murdered by patients or their relatives in hospitals and clinics across the country [43], which led Chinese doctors of the new generation feels lost. The participants believed that, the fear of potential violence might negatively influence practitioners’ sharing of clinical information, and might lead to a lack of high-quality medical information online. In that case, all the medical major students had never done online help-seeking, for they perceived not only waiting for responses from strangers was a waste of time, but also the quality of the answer was uncertain.

All the interviewees of the medical major agreed that people should try their best to protect the privacy of personal information and choose those tools ran by responsible companies. Although it was hard, those medical students insisted that avoiding information leakage should be treated seriously, with smart strategies, such as posting as little personal information as possible on strangers’ network, setting a blocked list for those untrustworthy netizens or even friends, setting the viewable scope of the posts, and selecting quality eHealth servicers to avoid information leakage. Moreover, the medical major group strongly believed that the government should make an effort to better manage the internet environment and should require companies to take the responsibility of information protection. Guidance for overcoming online scams and online stalking was also suggested to be provided by school or the government.

## Discussion

The primary aim of this research was to profile Chinese college students’ eHealth usage behaviors. A purposive sample was chosen to provide a comprehensive picture of eHealth usage behaviors, including five fundamental elements: information obtaining, information evaluating, online socializing, information applying and risk-handling. These elements differed between students of different college majors. Their differences were profiled with three main themes, i.e., expectance, usage pattern and perception. Few studies have compared eHealth usage behaviors among students studying with different majors in college. Only two studies had similar investigation in which college major was found to be a significant factor for effective eHealth usage [29, 30]. Their findings were in line with the results of the current research. The secondary purpose of the current research was to define eHealth literacy (required capabilities on eHealth usage) in the context of internet environment in the present time. Based on the eHealth usage behaviors derived in this study, the related skills were identified using the trilogy of Web 1.0 to 3.0, and derived a conceptual framework for eHealth literacy in the present day. Specifically, the Web 1.0 related eHealth literacy includes the capabilities of problem identifying, tool choosing, searching, filtering, cross-checking, distinguishing, appraising and decision-making; the Web 2.0 related eHealth literacy includes the capabilities of problem describing, responding, information communicating, peer competing, posting, sharing and online critics handling; the Web 3.0 related eHealth literacy includes the capabilities of self-data recording, self-tracking, self-managing, risk evaluating, danger avoiding, personal information protecting and internet order preserving.

Importantly, the current research found that the differed eHealth usage of different major students can be explained by the Theory of Planned Behavior (TPB) [53, 54], a classical behavior change theory. The current study showed that, first, different attitude toward eHealth-based communication and eHealth tools led to different level of using eHealth tools intention; second, the perceived social pressure influenced students’ using intention as well; third, students had stronger intention when they had better perceived behavioral control or confidence in evaluating and applying eHealth information; and fourth, students with stronger intention reported higher frequency of eHealth tools usage and better strategies for effective utilization. The current research has shown that the usage of eHealth tools could be considered as a kind of health behavior, TPB could be used to explain an individual’s eHealth tools usage. Attempts to construct or tailor interventions for college students or individuals with limited intention or abilities of eHealth tools usage can be explored.

This study provided a first look at the web 3.0 related skills, and delved deeper into the eHealth usage of social network services, suggested that the content of eHealth literacy should be updated and go beyond of ‘web-based’ ‘literature-review-like’ skills. Previous definitions of eHealth literacy have been outdated because most of them were based on the old Web 1.0 environment [32, 35, 55–58] and a few were on the less than current Web 2.0 one [59, 60]. But nowadays, with the evolution of technology, the continued connection with and dependence on cellphone have increased significantly, more and more eHealth services are provided on Apps and SNSs [18]. New competencies in usage of eHealth tools were required by these trendy eHealth services [23, 33, 34], specifically, the skills of effectively using Web 2.0 and 3.0 tools. Additionally, compared to eHealth information searching and evaluation (Web 1.0 related skills), making use of nowadays’ eHealth information seems to become a more important issue for health management and maintenance. New technologies provide more personalized eHealth services than before and change the context of internet. People perceived more convenient to obtain health information or create their own health data online, but there are certain thresholds for individuals to dig deeper and make full use of the obtained information. This study offered an updated definition of eHealth literacy, which can bring us new understanding on how we interact with nowadays eHealth tools and what kind of capabilities we should possess for these tools.

Meanwhile, unforeseen patterns of eHealth-based communications were found, reflected that the e-culture today is more interactive and complicate. For instance, the flamers and the trolls appeared on the eHealth forums or applications gave a negative influence to the e-culture, but not many internet users knew how to disarm them. The pre-selected personalized data-push technology also limited the diversity of information content exposed to people and build up an information cocoon [58]. The current research suggests that workshops on eHealth tools usage or eHealth literacy with the consideration of nowadays e-environment are worth to be provided among college students. New competencies such as critics handling, critical thinking and diversified eHealth access should be included in these workshops. Future research or intervention on this issue is warranted.

Furthermore, eHealth industry will be the most promising industry in the twenty-first century. Chinese eHealth industry has been booming as a rapidly growing sector with an estimated year-on-year growth rate of 29% [59]. Such rapid boom of the industry is happening all over the world. Thus, it is essential to promote guidance for eHealth usage to the public. Workshop should be organized by mass media and influencers to educate the public more about eHealth usage skills. Reliable eHealth websites could be promoted to the public by health-related governmental organizations. Additionally, it can not only deliver some high-quality eHealth information, but also provide positive social support for eHealth usage.

### Limitations and strengths

The limitations of this paper were that, the results were only based on the interviews among Chinese college students, therefore its application in other groups or from other geographical areas is needed to be examined. In addition, use of technology is ever changing with the technology development, thus the validity of the findings may be sensitive to the technological change. It is believed that further research should be conducted if the changing IT environment required, so that practitioners and users would be able to keep abreast of the times.

In spite of these limitations, the current study has recruited participants from different Chinese cities and different majors to avoid the influence of health disparity and improve the sample’s representation. An understanding of eHealth usage was also achieved in the context of Web 3.0 and Chinese culture. Moreover, the finding that students from different majors used eHealth in different ways may provide new and valuable information for researchers and educators to construct eHealth literacy training in the future. In addition, the online eHealth interpersonal interactivities nowadays had not been well explored yet. This study may shed light on this area and provide more in-depth and comprehensive information for further eHealth communication and behavior research. In the long term, the findings may also benefit eHealth service providers to develop the existing eHealth literacy tools, and for practitioners who are planning to develop strategies to promote eHealth in China.

## Conclusion

This paper provided an overview on nowadays eHealth usage among Chinese college student, then profiled and compared the expectation, the usage pattern and the perception of eHealth among students from different majors. A cutting-edge understanding of the eHealth literacy in nowadays IT environment was also obtained on the basis of the investigation on students’ eHealth usage. A first look at web 3.0 related eHealth behaviors was presented, found that individuals nowadays could create their own health data other than dealing with the existing eHealth information, the application of information becomes more complex and important than before, and education background may strongly shape people’s eHealth usage from the very beginning (expectance) to the very end (perception). The findings may be beneficial for further eHealth-related studies to better understand people’s eHealth usage, and may provide information for eHealth services providers and policy makers in this area.

## Data Availability

The datasets used and/or analyzed during the current study are available from the corresponding author on reasonable request.

## Footnotes

1 It refers to a piece of news in 2014, that a dying Chinese student’s family accused Baidu, the top search engine in China, to promoting false medical information and unqualified overpriced Chinese private hospital – the Putian Hospital. This news had led to a trend of distrust on online eHealth information.
